# Assessing the impact of different penalty factors of the Bayesian reconstruction algorithm Q.Clear on in vivo low count kinetic analysis of [^11^C]PHNO brain PET-MR studies

**DOI:** 10.1186/s13550-022-00883-1

**Published:** 2022-02-20

**Authors:** Daniela Ribeiro, William Hallett, Oliver Howes, Robert McCutcheon, Matthew M. Nour, Adriana A. S. Tavares

**Affiliations:** 1 Invicro, Centre for Imaging Sciences, Hammersmith Hospital, Invicro, Imperial College London, Burlington Danes Building, Du Cane Road, London, W12 0NN UK; 2 Edinburgh Imaging, The University of Edinburgh, Edinburgh, UK; 3 Institute of Psychiatry, Psychology and Neuroscience, King’s College London, London, UK; 4 Institute of Medical Sciences, Medical Research Council London, London, UK; 5 Institute of Clinical Sciences, Faculty of Medicine, Imperial College London, London, UK; 6 Max Planck Centre for Computational Psychiatry and Ageing Research, Institute of Neurology, University College London, London, UK; 7 Wellcome Centre for Human Neuroimaging, Institute of Neurology, University College London, London, UK

**Keywords:** PET-MR, [^11^C]PHNO, Reconstruction, Bayesian, Neuroimaging

## Abstract

**Introduction:**

Q.Clear is a Bayesian penalised likelihood (BPL) reconstruction algorithm available on General Electric (GE) Positron Emission Tomography (PET)-Computed Tomography (CT) and PET-Magnetic Resonance (MR) scanners. This algorithm is regulated by a *β* value which acts as a noise penalisation factor and yields improvements in signal to noise ratio (SNR) in clinical scans, and in contrast recovery and spatial resolution in phantom studies. However, its performance in human brain imaging studies remains to be evaluated in depth. This pilot study aims to investigate the impact of Q.Clear reconstruction methods using different *β* value versus ordered subset expectation maximization (OSEM) on brain kinetic modelling analysis of low count brain images acquired in the PET-MR.

**Methods:**

Six [^11^C]PHNO PET-MR brain datasets were reconstructed with Q.Clear with *β*100–1000 (in increments of 100) and OSEM. The binding potential relative to non-displaceable volume (*BP*_*ND*_) were obtained for the Substantia Nigra (SN), Striatum (St), Globus Pallidus (GP), Thalamus (Th), Caudate (Cd) and Putamen (Pt), using the MIAKAT™ software. Intraclass correlation coefficients (ICC), repeatability coefficients (RC), coefficients of variation (CV) and bias from Bland–Altman plots were reported. Statistical analysis was conducted using a 2-way ANOVA model with correction for multiple comparisons.

**Results:**

When comparing a standard OSEM reconstruction of 6 iterations/16 subsets and 5 mm filter with Q.Clear with different *β* values under low counts, the bias and RC were lower for Q.Clear with *β*100 for the SN (RC = 2.17), Th (RC = 0.08) and GP (RC = 0.22) and with *β*200 for the St (RC = 0.14), Cd (RC = 0.18)and Pt (RC = 0.10). The p-values in the 2-way ANOVA model corroborate these findings. ICC values obtained for Th, St, GP, Pt and Cd demonstrate good reliability (0.87, 0.99, 0.96, 0.99 and 0.96, respectively). For the SN, ICC values demonstrate poor reliability (0.43).

**Conclusion:**

*BP*_*ND*_ results obtained from quantitative low count brain PET studies using [^11^C]PHNO and reconstructed with Q.Clear with *β* < 400, which is the value used for clinical [^18^F]FDG whole-body studies, demonstrate the lowest bias versus the typical iterative reconstruction method OSEM.

**Supplementary Information:**

The online version contains supplementary material available at 10.1186/s13550-022-00883-1.

## Introduction

Positron Emission Tomography (PET) is an imaging technique that allows for non-invasive quantitative measurement of biological processes in vivo. Filtered Back Projection (FBP) has been used as the preferred reconstruction method in dynamic quantitative brain PET imaging research due its linearity, robustness and reliable results however Ordered Subset Expectation Maximisation (OSEM) is often used for semi-quantitative clinical whole-body and brain imaging due to its ability to provide better image quality [1]. FBP is not available in recently developed scanners, including the General Electric (GE) Signa PET- Magnetic Resonance (MR) scanners and therefore other alternatives have been devised. Current reconstruction algorithms such as OSEM and Block Sequential Regularised Expectation Maximisation (BSREM) are considered iterative reconstruction algorithms and can be used in images acquired in PET-Computed Tomography (CT) and in PET-MR scanners [2]. Previous studies [3–5] conducted in PET-CT scanners have demonstrated that OSEM presents better image quality and signal to noise ratio than FBP, therefore making it a suitable alternative to be used in clinical brain studies acquired in the PET-MR scanner. The suitability of BSREM algorithms in this setting has however not been extensively explored. Moreover, it has been shown that results obtained from OSEM reconstructions are biased in low statistics and it is unclear if BSREM algorithms perform in the same way [1].

The BSREM algorithm is a Bayesian penalised likelihood (BPL) reconstruction algorithm that uses prior knowledge as a penalty term in the iterative process. The *β* value (editable parameter in the algorithm) regulates the strength of the penalty term, acting as a noise penalisation factor and improves the Signal to Noise ratio (SNR). GE Healthcare has released the BSREM penalised likelihood reconstruction algorithm with the denomination of Q.Clear [6, 7]. PET images can be analysed with qualitative methods, which are based on visual assessments, and semi-quantitative or quantitative methods, such as standard uptake values or volumetric measurements, respectively [8]. The literature regarding the use of Q.Clear as a reconstruction algorithm for quantification is limited, with some manuscripts investigating the effect of the algorithm in phantom images [7, 9, 10]. Most of the available literature is primarily focused on fluorinated tracers, with some publications investigating the effect of the algorithm in semi-quantification of whole-body scans and/or small structures imaging [11–14]. Furthermore, there is limited knowledge on the quantitative accuracy of Q.Clear when reconstructing brain PET-MR images with low counts and high noise.

[^11^C]PHNO is a PET radiotracer that binds to both D2 and D3 dopamine receptors which are part of the D2-like dopaminergic receptors (DARs) family [15, 16]. Unlike antagonist radiopharmaceuticals, agonist radiotracers such as [^11^C]PHNO have the potential to produce pharmacologic effects [17, 18]. In practice, a compromise between mass and activity must be reached before the scan, in order to avoid side effects, and it is sometimes necessary to administer an activity much lower than the target activity [18]. The restricted injected dose limits may result in noisy imaging data with low counts. Moreover, for studies that require multiple scans, for example for longitudinal follow-up or to investigate the effects of drug challenges [19], it is necessary to limit the injected dose to ensure the total radiation dose remains within an acceptable range for research. In these circumstances it is particularly important to use a reconstruction algorithm that maximises the SNR.

Image reconstruction algorithms may have an impact on measured binding potential relative to non-displaceable volume measurements *(BP*_*ND*_) calculated when using a simplified reference tissue model (SRTM), although this has not been fully assessed with the latest reconstruction methods, such as Q.Clear [20]. Hence, we aim to investigate the impact of Q.Clear reconstruction methods on brain kinetic modelling analysis, which will provide new knowledge compared with previously conducted studies focused on characterising simplified outcome measure bias (e.g. standard uptake value (SUV)) introduced by Q.Clear reconstruction methods. The primary objective of this pilot study is to investigate the performance of Q.Clear, against the performance of the established OSEM algorithm, in low activity [^11^C]PHNO PET brain images acquired on a PET-MR system. We also investigate which Q.Clear *β* values provides similar quantitative results for low count brain scans, to those observed with a OSEM 6 iteration, 16 subset and 5 mm filter reconstruction (which is a routinely used clinical standard reconstruction for brain PET-MR scans, including within our department). This will provide important evidence to the field, given that previous work has been predominantly focused on the use of Q.Clear methods for reconstruction of whole-body PET data and routine non-kinetic modelling studies.

## Materials and methods

### [^11^C]PHNO PET-MR human data acquisition and reconstruction

The original study adhered to the principles outlined in the National Health Service (NHS) Research Governance Framework for Health and Social Care (2nd edition), the Declaration of Helsinki and Good Clinical Practice (GCP). It was also conducted in compliance with the Protocol, the Data Protection Act and other regulatory requirements, and Standard Operating Procedures (SOPs), as appropriate. The data that were used in this project were acquired after the participant’s consent was obtained for the original study (REC reference 12/LO/1955, IRAS Project ID: 103938). Use of this data was covered in the original consent form, which stated that the data acquired could be used in future related research.

Seven in vivo [^11^C]PHNO PET datasets, corresponding to seven different healthy normal participants, were reconstructed retrospectively using Q.Clear and OSEM algorithms, for this pilot study. The average age of the participants was 23 years with the female to male ratio being 3:4. The mean administered dose was 145.8 ± 15.8 MBq (mean ± SD, *n* = 7).

An MRI-based attenuation correction (MRAC) sequence (MRI sequence with flip angle of 5°, echo time (TE) 1.674 ms, repetition time (TR) 4.048 ms, 50 × 38 cm FOV with 256 × 128 matrix), which was obtained during scan acquisition, was used for attenuation correction of the PET data.

Typical [^11^C]PHNO PET-MR scans were binned into the following frames 10 × 15 s, 3 × 60 s, 5 × 120 s, 15 × 300 s, with a total duration of 90 min and 30 s. The dataset was processed once with the above frames and with a reconstruction of OSEM 6iterations, 16subsets and a 5 mm Gaussian filter, with time of flight (TOF) information. This was entitled “26_OSEM_6i16s5mm_normal”. In order to mimic a low count acquisition, the dynamic PET-MR scans were reprocessed with a pre-frame delay thereby decreasing the time per frame by a factor of 3. Each in vivo dataset was reconstructed 11 times (10 TOF Q.Clear reconstructions [with *β* between 100 and 1000, in increments of 100], and 1 TOF OSEM reconstruction [6iterations, 16subsets and a 5 mm Gaussian filter]), with the pre-frame delay and named with the suffix “_low”. Normal [^11^C]PHNO scans present an average count level of 4.9 × 10^7^ counts at the 15-min frame, 1.1 × 10^7^ counts at the 45-min frame and 2.6 × 10^6^ counts at the 90-min frame. When simulating a low dose acquisition, the 15-min frame presented an average count level of 1.5 × 10^7^ counts, the 45-min frame 3.3 × 10^6^ counts and the 90-min frame 8.3 × 10^5^ counts. Additional file 1: Figure S1 contains a graphic of the prompt events over time, for the normal and low count datasets, for one participant. For ease of comparison, the European Association of Nuclear Medicine advises that, for static brain [^18^F]FDG scans, 100 million events should be detected for a duration of 10–20 min [21]. The scan reconstructed with OSEM 6iterations, 16subsets and a 5 mm Gaussian filter under normal counts was only used for the comparison with its counterpart under low counts, to establish the extent of the variability when using the same reconstruction parameters and different count statistics. Point Spread Function (PSF) modelling was not used for the OSEM or Q.Clear reconstructions (PSF modelling is included in Q.Clear by default) and all datasets were reconstructed using time of flight information.

### Data analysis

All reconstructed [^11^C]PHNO dynamic human brain PET scans were run through the MIAKAT™ (www.miakat.org) pipeline in order to obtain *BP*_*ND*_ results for the Substantia Nigra (SN), Striatum (St), Globus Pallidus (GP), Thalamus (Th), Caudate (Cd) and Putamen (Pt). The pipeline in MIAKAT™ follows a sequence of steps namely, Brain Extraction, Brain Tissue Segmentation, Motion Correction, Region of interest (ROI) definition, ROI tracer kinetic modelling and Parametric imaging. Motion correction and ROIs were applied to all reconstructions for the same subject. No image processing was performed prior to the datasets being run through MIAKAT™, however the outputs from the steps described above were reviewed and manually accepted by the investigator. The data analysis steps required limited interaction from the investigator and the data analysis process for all images datasets was conducted by the same investigator, hence reducing intra-operator and inter-operator variability. Since a region devoid of receptors was available, i.e. the cerebellum, it was possible to use a SRTM approach to estimate *BP*_*ND*_, which is a product of the receptor density and affinity and provides information regarding non-specific and free radioligand concentrations [22].

Intraclass Correlation Coefficient (ICC) estimates and 95% confident intervals (CI) were calculated using SPSS statistical package version 26 (SPSS Inc, USA) based on 11 reconstruction items (TOF_OSEM_6i16s5mm_low, TOF_QClear_B100_low, TOF_QClear_B200_low, TOF_QClear_B300_low, TOF_QClear_B400_low, TOF_QClear_B500_low, TOF_QClear_B600_low, TOF_QClear_B700_low, TOF_QClear_B800_low, TOF_QClear_B900_low and TOF_QClear_B1000_low), absolute-agreement, 2-way mixed-effects model.

Bland–Altman plots were obtained with GraphPad Prism version 8.0.0 for Windows (GraphPad Software, USA). Bias and the Repeatability Coefficient (RC) between the OSEM algorithm (6iterations, 16subsets, 5 mm filter reconstruction under low counts, defined as standard reconstruction for the purposes of this study) and the Q.Clear reconstructions (*n* = 10, with differing *β* values), were produced using MedCalc® version 18 (MedCalc Software, Belgium), computing the standard deviation of the BP_ND_ results obtained for the healthy subjects. The 2-way ANOVA results and multi comparisons using the Bonferroni test were used to determine group differences among *BP*_*ND*_ results for the SN, St, GP, Th, Cd and Pt groups for the in vivo data. For this purpose, for determining the Coefficients of Variation (CV) and for graphical demonstration, GraphPad Prism version 8.0.0 for Windows (GraphPad Software, USA) was used.

## Results

**Fig. 1:**
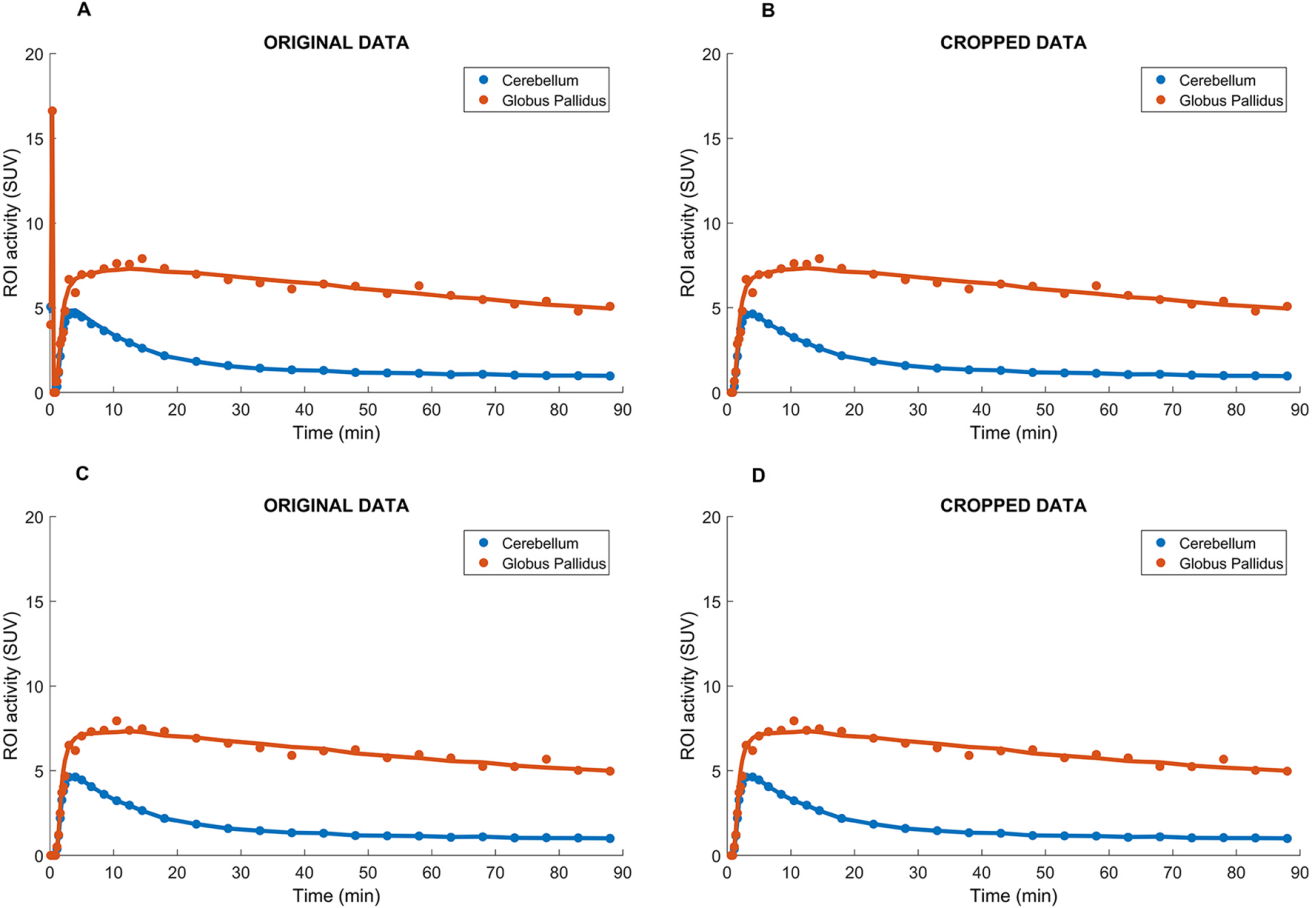
Model fitting obtained for the Cerebellum and Globus Pallidus, one PET-MR brain dataset (same subject) reconstructed with TOF Q.Clear *β*100 (top row) and OSEM (bottom row). Note the interference of the background counts on the model fitting on the graphic entitled “Original Data” (**A**). The three initial frames that contained background counts were removed on the graphic entitled “Cropped data” (**B**). Note the lack of interference from the background counts, when OSEM is used, on the model fitting on graphic (**C**) and the similar model fitting obtained when the initial frames are removed for the OSEM reconstructed, on graphic (**D**)

Out of the seven initial in vivo datasets only six were used for the statistical analysis due to one dataset presenting excessive movement that could not be corrected during image processing. It was noted that the first frames of the Q.Clear reconstructions presented spurious counts that did not correspond to the radiopharmaceutical injection, interfering with the time activity curves (TACs) and the kinetic modelling analysis. As the injection was only administered 30 s after the start of the acquisition, these frames were devoid of radioactivity. After removing the first three frames from the reconstructed images, the curves obtained presented the expected behaviour. An example of the model fitting for the Globus Pallidus and Cerebellum, for the same subject, when the brain images were reconstructed with Q.Clear with *β*100 and OSEM can be found in Fig. 1. The graphics entitled “original data” (A) and (C) demonstrate the fit obtained with all the frames included. The graphic entitled “cropped data” (B) and (D) demonstrate the fit obtained when the 3 first frames were removed hence removing the background counts that did not correspond to the radiopharmaceutical injection. Graphics (A) and (B) correspond to the data reconstructed with Q.Clear *β*100 whereas graphics (C) and (D) correspond to the data reconstructed with OSEM.

**Fig. 2:**
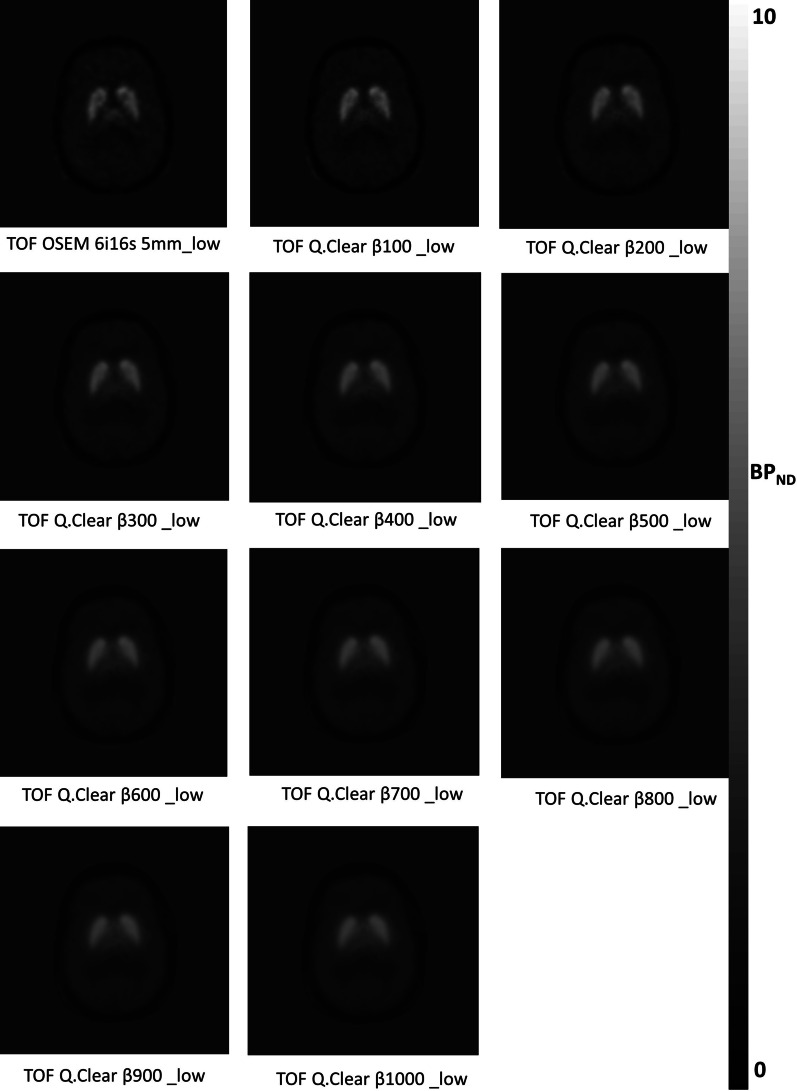
Representative *BP*_*ND*_ parametric brain images after [^11^C]PHNO administration, per reconstruction method under low counts. Note the visual differences in image quality for the Q.Clear reconstructions as *β* increases

Example images of the [^11^C]PHNO *BP*_*ND*_ obtained for one participant from the in vivo dataset and reconstructed with standard OSEM and Q.Clear with *β*100–1000 are present in Fig. 2.

For large brain regions, such as the thalamus and the striatum, the intraclass correlation coefficient analysis demonstrates that there is a good reliability, with the ICC obtained for the *BP*_*ND*_ results being 0.87 (95% CI 0.70–0.98) for the thalamus and 0.99 (95% CI 0.97–0.99) for the striatum.

**Fig. 3:**
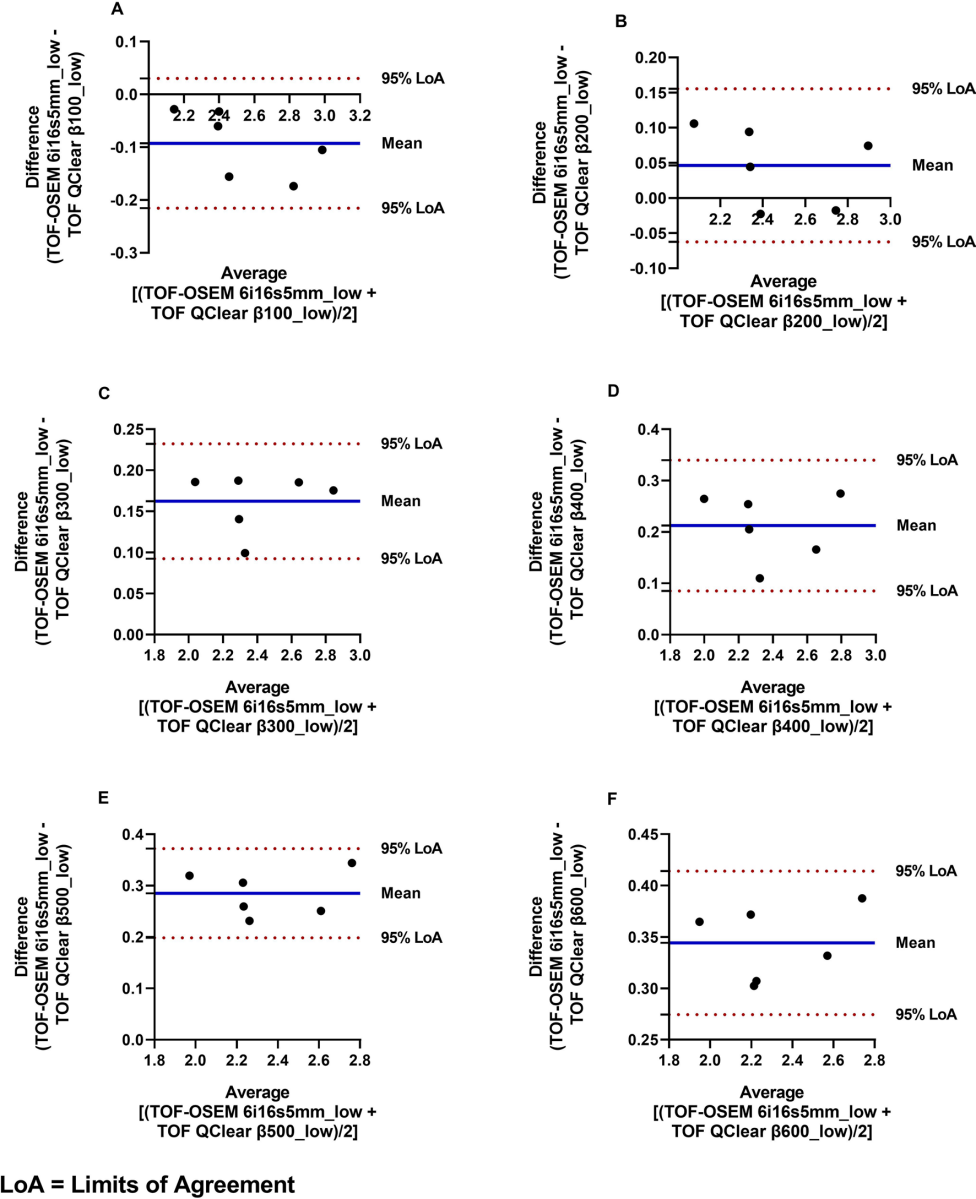
Bland–Altman plots of the *BP*_*ND*_ obtained for the Striatum: **A** TOF OSEM 6i16s5mm_low versus TOF Q.Clear β100_low; **B** TOF OSEM 6i16s5mm_low versus TOF Q.Clear β200_low; **C** TOF OSEM 6i16s5mm_low versus TOF Q.Clear β300_low; **D** TOF OSEM 6i16s5mm _low versus TOF Q.Clear β400_low; **E** TOF OSEM 6i16s5mm_low versus TOF Q.Clear β500_low; **F** TOF OSEM 6i16s5mm_low versus TOF Q.Clear β600_low

**Fig. 4:**
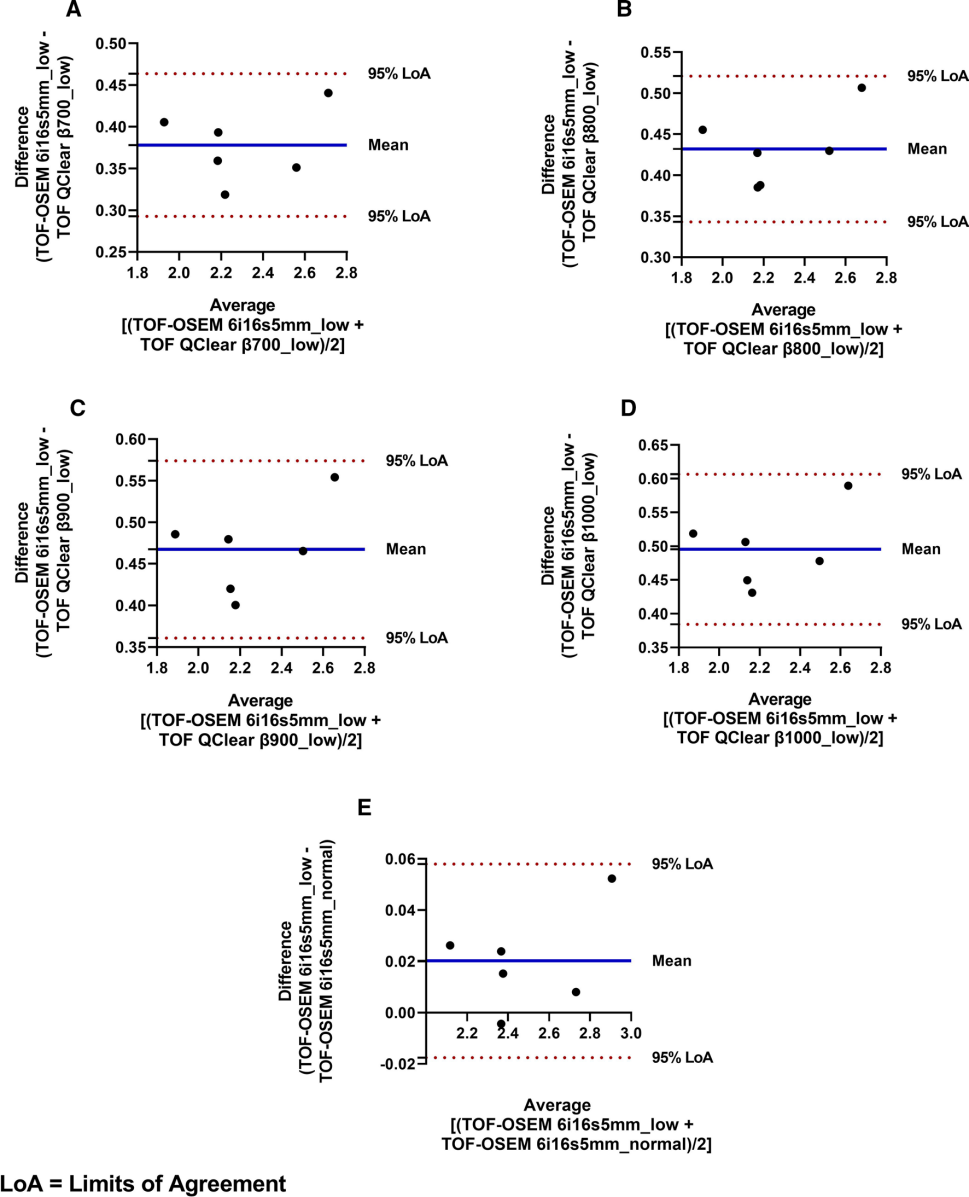
Bland–Altman plots of the *BP*_*ND*_ obtained for the Striatum: **A** TOF OSEM 6i16s5mm _low versus TOF Q.Clear β700_low; **B** TOF OSEM 6i16s5mm _low versus TOF Q.Clear β800_low; **C** TOF OSEM 6i16s5mm_low versus TOF Q.Clear β900_low; **D** TOF OSEM 6i16s5mm_low versus TOF Q.Clear β1000_low; **E** TOF OSEM 6i16s5mm _low versus TOF OSEM 6i16s5mm_normal

For the Thalamus, when comparing with the standard reconstruction, the Q.Clear with *β*100 reconstruction presented the lowest bias (0.002) and RC (0.08). The full bias and RC results are present in Additional file 6: Table S1. In the Striatum, the Q.Clear with *β*200 reconstruction presents the lowest bias (0.046) and RC (0.14), when compared to the standard reconstruction. This is demonstrated in the Bland–Altman plots of Figs. 3 and 4.

**Fig. 5:**
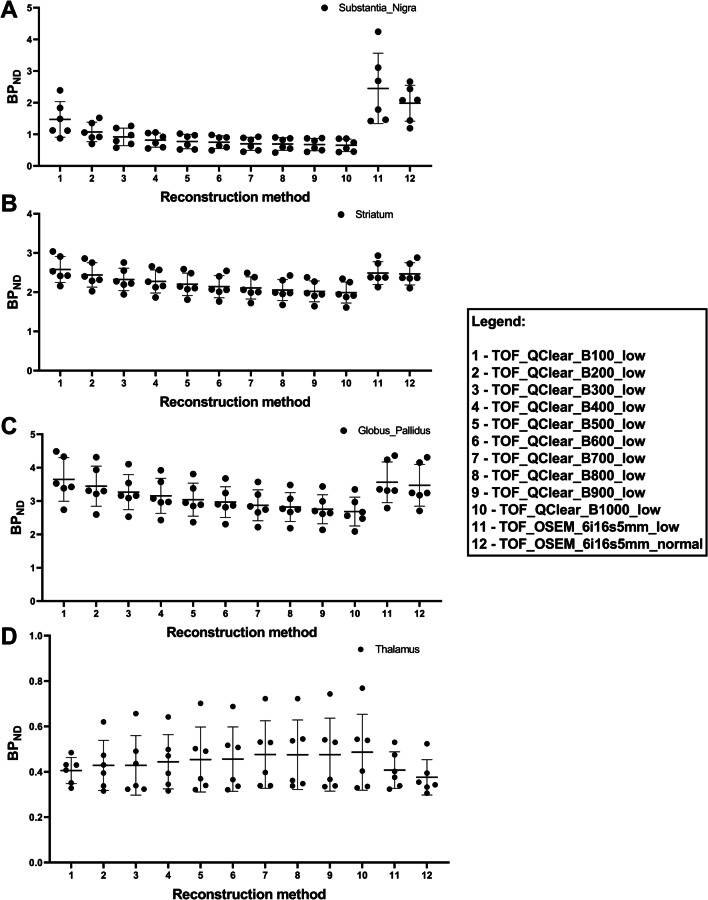
Graphic layout of the *BP*_*ND*_ obtained for the Substantia Nigra (**A**), Striatum (**B**), Globus Pallidus (**C**) and Thalamus (**D**), per reconstruction method. For the Substantia Nigra (**A**), Striatum (**B**) and Globus Pallidus (**C**) as the *β* value for the Q.Clear reconstructions increases, the mean *BP*_*ND*_ decreases. However, for the Thalamus (**D**), as the β value for the Q.Clear reconstructions increases, the mean *BP*_*ND*_ increases

A graphic layout of the *BP*_*ND*_ obtained for the Substantia Nigra (A), Striatum (B), Globus Pallidus (C) and Thalamus (D), per reconstruction method is presented in Fig. 5.

For medium size brain regions, such as the Globus Pallidus, Putamen and Caudate the intraclass correlation coefficient analysis demonstrates that there is a good reliability (with the ICC obtained for the *BP*_*ND*_ results being 0.96 (95% CI 0.89–0.99), 0.99 (95% CI 0.98–0.99) and 0.96 (95% CI 0.90–0.0.99) respectively. In the Globus Pallidus, *BP*_*ND*_ data shows that Q.Clear with *β*100 reconstruction presented the lowest bias (− 0.087) and RC (0.22), when compared to the standard reconstruction. This is demonstrated in the Bland–Altman plots for the Globus Pallidus in the Additional file 2: Figure S2 and Additional file 3: Figure S3. The results for the Caudate and Putamen demonstrate a similar pattern to what was observed for the structures in graphs A, B and C in Fig. 5. When compared to the standard reconstruction, Q.Clear with *β*200 reconstruction presented the lowest bias and RC for both the Cd and Pt (bias of − 0.041 and 0.015 and RC of 0.18 and 0.10, respectively).

For small size brain regions, namely the Substantia Nigra, the intraclass correlation coefficient analysis demonstrated poor intra-rater reliability (the ICC obtained for the *BP*_*ND*_ results for the SN was 0.43 (95% CI 0.17–0.83). The Q.Clear reconstruction with *β*100 presented the lowest bias (0.979) and RC (2.17), when compared to the standard OSEM reconstruction. This is demonstrated in the Bland–Altman plots for the Substantia Nigra in the Additional file 4: Figure S4 and Additional file 5: Figure S5.

The *BP*_*ND*_ results in the Substantia Nigra for the OSEM 6 iterations, 16 subsets and filter of 5 mm reconstruction mimicking low counts were more dispersed (CV = 45.42%) than the results for the same reconstruction with a normal number of counts (CV = 28.61%) and the comparison between both datasets provided a bias of 0.469 and RC of 1.49. For all other brain regions, the dispersion was similar for both the normal counts and the reduced counts reconstructions. The full list of percentage CV is present in Additional file 7: Table S2.

**Fig. 6:**
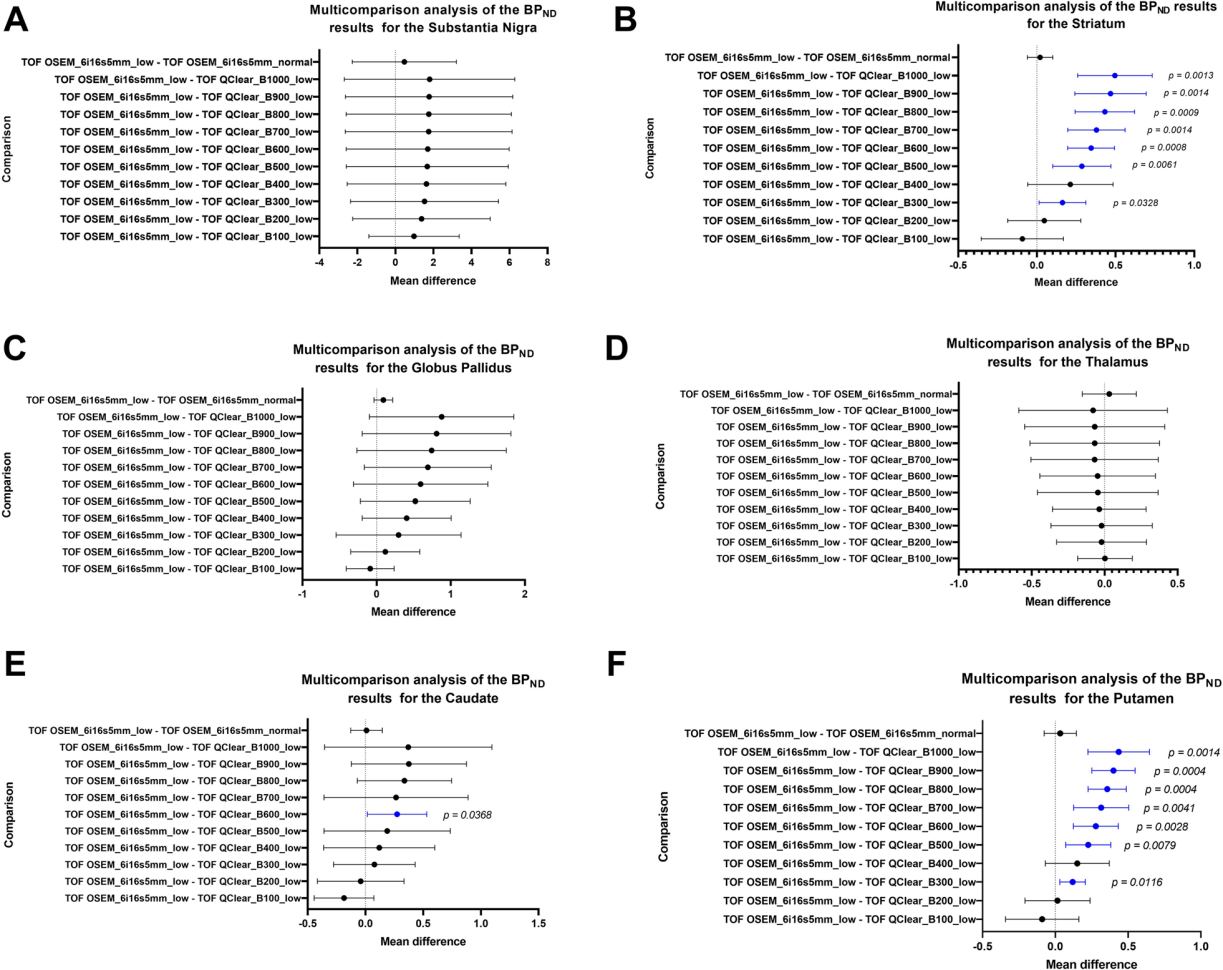
Multicomparison analysis of the *BP*_*ND*_ results obtained for all structures when images reconstructed the standard OSEM 6iterations 16subsets and 5 mm filter and with the Q.Clear reconstructions with different *β* values. Note the statistically significant results included on the graphs

When comparing the *BP*_*ND*_ results from the standard iterative reconstruction, OSEM with 6 iterations, 16 subsets and a filter with 5 mm kernel under low counts, with the Q.Clear reconstructions with different *β* values under low counts, for the Substantia Nigra, Globus Pallidus and Thalamus, there is no comparison that provides a p-value that is statistically significant. Conversely, the Q.Clear with a *β*300, 600, 800 and 900 showed statistically significant differences, when compared to OSEM with 6 iterations, 16 subsets and a filter with 5 mm kernel, for the Striatum and Putamen (Fig. 6).

## Discussion

The aim of this study was to investigate the impact of Q.Clear reconstruction methods on brain kinetic modelling analysis by evaluating the performance of Q.Clear, against the performance of OSEM in the presence of a small number of counts, in brain images acquired in a PET-MR system. To our knowledge, we are the first to investigate Q.Clear reconstruction performance for brain kinetic modelling analysis rather than simplified quantification methods like standard uptake value (SUV). We also report here that, for low count brain scans in comparison to whole-body PET imaging, much lower *β* levels (between 100 and 200) are required to achieve the same quantitative results to those obtained with a OSEM method.

The results for all structures, apart from the Substantia Nigra, appeared to be unaffected by the reconstruction method as the changes in the CV were minimal. The Substantia Nigra however appeared to be vulnerable to the reconstruction method under a low count scenario as not only the results appeared more dispersed, but it was also observed an increase of almost 12% for the CV calculation. This finding demonstrates that, when conducting kinetic modelling with an SRTM, the reconstruction algorithm used may have a different impact on different brain structures. This project did not consider partial volume effect correction which is important for small structures such as the Substantia Nigra. Even though Q.Clear improves spatial resolution versus OSEM due to PSF corrections, this is still limited and a consequence of it is the partial volume effect which can affect the PET images quantitatively [9]. Therefore, the results for the Substantia Nigra could be underestimated by a spill-over effect from the white matter located in the midbrain [23].

The penalisation factor in Q.Clear performs as a noise suppression term with higher *β* values resulting in stronger noise reduction, whilst preserving edges [24]. This explains the decrease in the mean *BP*_*ND*_ results with the increase in *β* value, for the SN, St, and GP. The exception to this can be observed in the thalamus as there is a slight increase in the mean *BP*_*ND*_ results with the increase in *β* value, possibly due to the low target density in that region (with *BP*_*ND*_ values approximately 10 times lower than high density and large regions, such as the striatum).

The *BP*_*ND*_ obtained from the in vivo data demonstrates that, in a low count scenario, Q.Clear with *β*100 has the lowest bias when compared to the standard low count OSEM reconstruction for the SN, GP and Th. For the same metric in the Striatum, Q.Clear with *β*200 has the lowest bias. Furthermore, when the *BP*_*ND*_ for the Cd and Pt are investigated individually it is also noted that Q.Clear with *β*200 presents the lowest bias for both structures. These results are further substantiated by the multi comparison results which demonstrate that Q.Clear with *β*100, 200 and 400 are the only reconstructions across all structures that do not present statistically significant (0.01 < *p* ≤ 0.05), very statistically significant (0.001 < *p* ≤ 0.01) or extremely statistically significant (0.0001 < *p* ≤ 0.001) differences when compared to the standard reconstruction.

Lindström et al. (2017) investigated clinical whole-body scans which were acquired in a GE PET-CT system and reconstructed with Q.Clear and TOF-OSEM. They found that in order to obtain a noise equivalence to TOF-OSEM reconstructions with 3iterations, 16subsets and 5 mm Gaussian filter, a Q.Clear reconstruction with *β*600 should be performed for radiotracers such as ^68^ Ga-DOTATOC, ^18^F-FDG and ^18^F-Fluoride and a Q.Clear reconstruction with *β*400 should be performed for ^11^C-acetate [25].

Scott et al. (2019) aimed at optimising Q.Clear for ^90^Y quantitative imaging by preparing a National Electrical Manufacturers Association (NEMA) image quality phantom with an ^90^Y solution and scanning it on a GE Discovery 710 PET-CT scanner. Images were re-binned in the first instance into 15 min frames and, at a later stage, into 30 and 60 min frames and reconstructed with Q.Clear with *β* values of 1, 400, 800, 1000, 1200, 1400, 1600, 1800, 2000, 3000, 4000 and 8000. They calculated activity recovery and found that the optimal value for quantification was *β* 1000, as the reduction in image noise provided by this reduction does not affect quantification [26].

These reports demonstrate that the optimal *β* value is dependent on the tracer and the OSEM parameters used for a given application (e.g. brain PET studies versus whole-body PET). Notably, brain PET imaging requires more resolved images and this can be achieved with either an OSEM reconstruction with a high number of iterations and subsets or a Q.Clear reconstruction with low *β* values. It is encouraging that our results are in line with the report by Ross [27] who reconstructed two ^18^F-FDG brain image datasets with OSEM 3iterations, 32 subsets and 2.5 mm filter and Q.Clear with *β*150 and found that this *β* level produced excellent contrast and image quality in both datasets. Reynés-Llompart et al. (2018) evaluated phantom and brain and whole-body patient images which had been acquired in a GE Discovery IQ PET-CT system and reconstructed with Q.Clear with *β* from 50 to 500. They used various acquisition times to mimic different counts—the 15 s acquisition in their study yielded 19 ± 4 million counts, which represents the closest statistics to the ones mimicked in our study. At a 15 s acquisition and using a lesion to background ratio of 2:1, a *β* value of 150 maximises the contrast to noise ratio (CNR) for a sphere of 10 mm, a *β* value of 200 maximises the CNR for a sphere of 13 mm and a *β* value of 250 maximises the CNR for a sphere of 17 mm. Although in kinetic modelling spatial resolution is of more importance than CNR, it is important to note that *β* values of this range yield good contrast for small structures. Their results also demonstrated that for images of the torso, the optimal *β* value would be between 300 and 400, whereas for the brain images, it would be between 100 and 200, which is in line with our observation [28]. This suggests that, unlike diagnostic whole-body studies, using ^18^F-MISO and/or ^18^F-FAZA in hypoxic lung lesions [13] and ^18^F-FDG PET-CT in pulmonary nodules [10], where the optimal *β* value is 350 and 400 or studies using ^68^ Ga-PSMA and ^18^F-Fluciclovine in pelvic lesions [29, 30] which found that the optimal *β* value was between 400 and 550 and 300, respectively, for brain studies the optimal *β* value is lower, particularly when accurate quantification is paramount. In fact, phantom and clinical studies conducted with the aim of improving spatial resolution rather than for diagnostic purposes have reported that Q.Clear with low beta values provides better spatial resolution in small structures. Rogasch et al. (2020) investigated image metrics such as spatial resolution, contrast recovery and SNR in phantom images reconstructed with Q.Clear and OSEM with PSF modeling. The team found that when using Q.Clear with *β* 150 and a high signal to background ratio, the spatial resolution obtained is superior to that obtained when reconstructing images with PSF modelling and/or time of flight [9]. Similarly, a publication by Howard et al. [14] investigating Q.Clear in small pulmonary nodules reported that Q.Clear with a *β* value of 150 improved visual conspicuity of nodules of approximately 1 cm.

Our work follows a similar approach to that of Teoh et al., Ter Voert et al. and Teoh et al. [10, 29, 30]. However, whereas these investigations were performed in whole-body imaging and focusing on the effect of the algorithm on SUV metrics, our work was performed in quantitative dynamic brain imaging and demonstrates the effects on *BP*_*ND*_. To our knowledge, this has not been attempted before. Moreover, our work further sustains the initial observations presented by Reynés-Llompart et al. [28].

A limitation related with the use of Q.Clear that was noted in this study was that for frames with low counts, spurious high counts were seen in the reconstruction and three of the initial frames had to be removed (as was described in the Results section). This demonstrates the importance of the quality control stage in image analysis.

## Conclusion

In [^11^C]PHNO brain studies that require accurate quantification, Q.Clear with *β* values between 100 and 200 provide the least bias, lower RC and no statistically significant differences when compared to a standard OSEM reconstruction. Further investigations in this field are required to determine if *β* values in the range mentioned above provide the same results for other radiopharmaceuticals.

## Supplementary Information

**Additional file 1. Figure S1: **Graphic of the prompt events over time (time post-injection). Note the higher prompt counts for the plot denominated “normal”, which refers to the datasets with normal counts. The plot denominated “low” refers to the datasets in which low counts were simulated. Both plots belong to the same participant.

**Additional file 2. Figure S2: **Bland-Altman plots of the BPND obtained for the Globus Pallidus: (**A**) TOF_OSEM 6i16s5mm_low vs TOF_Q.Clear β100_low; (**B**) TOF_OSEM 6i16s5mm _low vs TOF_Q.Clear β200_low; (**C**) TOF_OSEM 6i16s5mm_low vs TOF_Q.Clear β300_low; (**D**) TOF_OSEM 6i16s5mm _low vs TOF_Q.Clear β400_low; (**E**) TOF_OSEM 6i16s5mm_low vs TOF_Q.Clear β500_low; (**F**) TOF_OSEM 6i16s5mm_low vs TOF_Q.Clear β600_low.

**Additional file 3. Figure S3: **Bland-Altman plots of the BPND obtained for the Globus Pallidus: (**G**) TOF_OSEM 6i16s5mm _low vs TOF_Q.Clear β700_low; (**H**) TOF_OSEM 6i16s5mm _low vs TOF_Q.Clear β800_low; (**I**) TOF_OSEM 6i16s5mm_low vs TOF_Q.Clear β900_low; (**J**) TOF_OSEM 6i16s5mm _low vs TOF_Q.Clear β1000_low; (**K**) TOF_OSEM 6i16s5mm _low vs TOF_OSEM 6i16s5mm_normal.

**Additional file 4. Figure S4: **Bland-Altman plots of the BPND obtained for the Substantia Nigra: (**A**) TOF_OSEM 6i16s5mm_low vs TOF_Q.Clear β100_low; (**B**) TOF_OSEM 6i16s5mm _low vs TOF_Q.Clear β200_low; (**C**) TOF_OSEM 6i16s5mm_low vs TOF_Q.Clear β300_low; (**D**) TOF_OSEM 6i16s5mm _low vs TOF_Q.Clear β400_low; (**E**) TOF_OSEM 6i16s5mm_low vs TOF_Q.Clear β500_low; (**F**) TOF_OSEM 6i16s5mm_low vs TOF_Q.Clear β600_low.

**Additional file 5. Figure S5: **Bland-Altman plots of the BPND obtained for the Substantia Nigra: (**G**) TOF_OSEM 6i16s5mm _low vs TOF_Q.Clear β700_low; (**H**) TOF_OSEM 6i16s5mm _low vs TOF_Q.Clear β800_low; (**I**) TOF_OSEM 6i16s5mm_low vs TOF_Q.Clear β900_low; (**J**) TOF_OSEM 6i16s5mm _low vs TOF_Q.Clear β1000_low; (**K**) TOF_OSEM 6i16s5mm _low vs TOF_OSEM 6i16s5mm_normal.

**Additional file 6. Table S1.** Bias, Standard deviation of Bias, Repeatability Coefficients (RC), Lower Limits of Agreement (LoA), Higher LoA, standard deviation of Bias and LoA obtained, per brain structure, when Q. Clear reconstructions with pre-frame delay and OSEM reconstruction with normal frame length were compared to standard OSEM reconstruction with pre-frame delay.

**Additional file 7. Table S2.** Coefficient of variation obtained for the Substantia Nigra, Striatum, Globus Pallidus, Thalamus, Caudate and Putamen, per reconstruction Method. Note the highest percentages are observed for the Substantia Nigra and Thalamus.

## Data Availability

The datasets generated and analysed during the current study are not publicly available due to proprietary restrictions but are available from the corresponding author on reasonable request.

## Abbreviations

BPL: Bayesian penalised likelihood
BPND: Binding potential relative to non-displaceable volume
BSREM: Block sequential regularised expectation maximisation
Cd: Caudate
CI: Confident intervals
CT: Computed tomography
CV: Coefficients of variation
DARs: D2-like dopaminergic receptors
FBP: Filtered back projection
GCP: Good clinical practice
GE: General Electric
GP: Globus pallidus
ICC: Intraclass correlation coefficient
MR: Magnetic resonance
MRAC: MRI-based attenuation correction
NEMA: National Electrical Manufacturers Association
NHS: National Health Service
OSEM: Ordered subset expectation maximisation
PET: Positron emission tomography
Pt: Putamen
RC: Repeatability coefficient
ROI: Region of interest
SN: Substantia Nigra
SNR: Signal to noise ratio
SOPs: Standard operating procedures
SRTM: Simplified reference tissue model
St: Striatum
TACs: Time activity curves
TE: Echo time
Th: Thalamus
ToF: Time of flight
TR: Repetition time

## Code availability

All analysis was done using custom made code at inviCRO, a Konica Minolta Company and is subject to proprietary restrictions on sharing. However, details on fundamentals of this software are described in the manuscript.
