# Supplementary figures and images for: Assessing the impact of different penalty factors of the Bayesian reconstruction algorithm Q.Clear on in vivo low count kinetic analysis of [11C]PHNO brain PET-MR studies
Source: EJNMMI Res. 2022 Feb 20;12:11. doi: 10.1186/s13550-022-00883-1 (PMC8859021; doi:10.1186/s13550-022-00883-1)

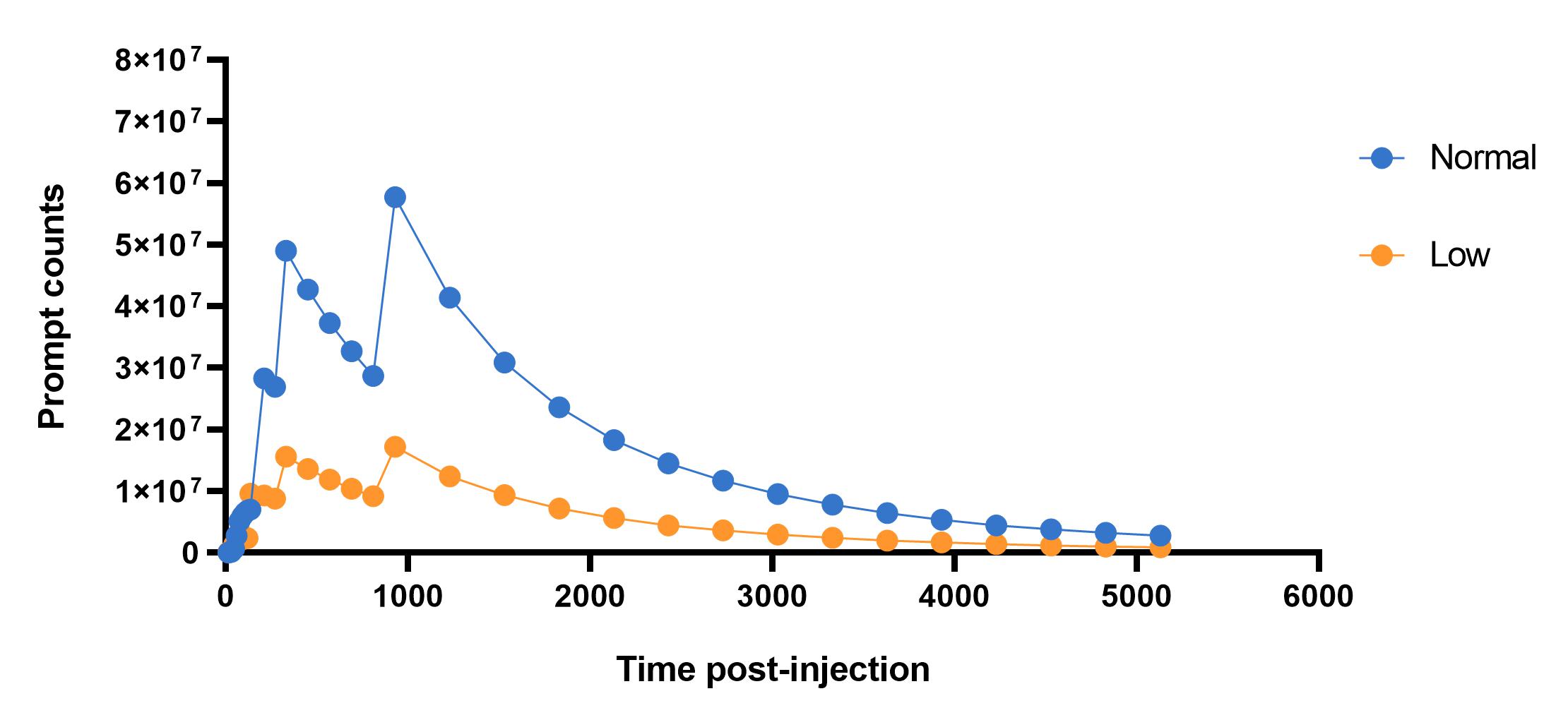

Supplement: Supplementary file 1 — Additional file 1. Figure S1: Graphic of the prompt events over time (time post-injection). Note the higher prompt counts for the plot denominated “normal”, which refers to the datasets with normal counts. The plot denominated “low” refers to the datasets in which low counts were simulated. Both plots belong to the same participant. [file 13550_2022_883_MOESM1_ESM.jpg]

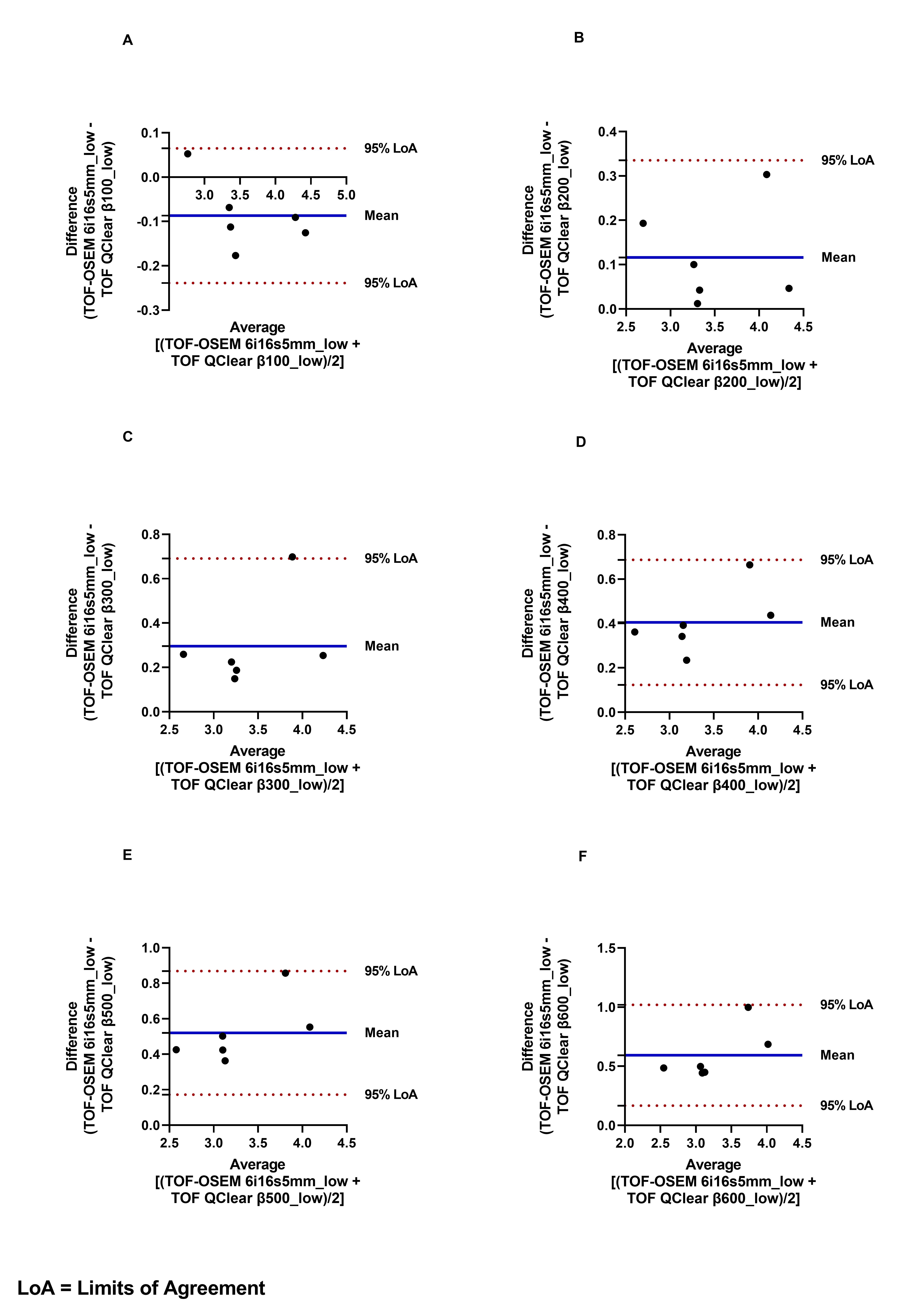

Supplement: Supplementary file 2 — Additional file 2. Figure S2: Bland-Altman plots of the BPND obtained for the Globus Pallidus: (A) TOF_OSEM 6i16s5mm_low vs TOF_Q.Clear β100_low; (B) TOF_OSEM 6i16s5mm _low vs TOF_Q.Clear β200_low; (C) TOF_OSEM 6i16s5mm_low vs TOF_Q.Clear β300_low; (D) TOF_OSEM 6i16s5mm _low vs TOF_Q.Clear β400_low; (E) TOF_OSEM 6i16s5mm_low vs TOF_Q.Clear β500_low; (F) TOF_OSEM 6i16s5mm_low vs TOF_Q.Clear β600_low. [file 13550_2022_883_MOESM2_ESM.jpg]

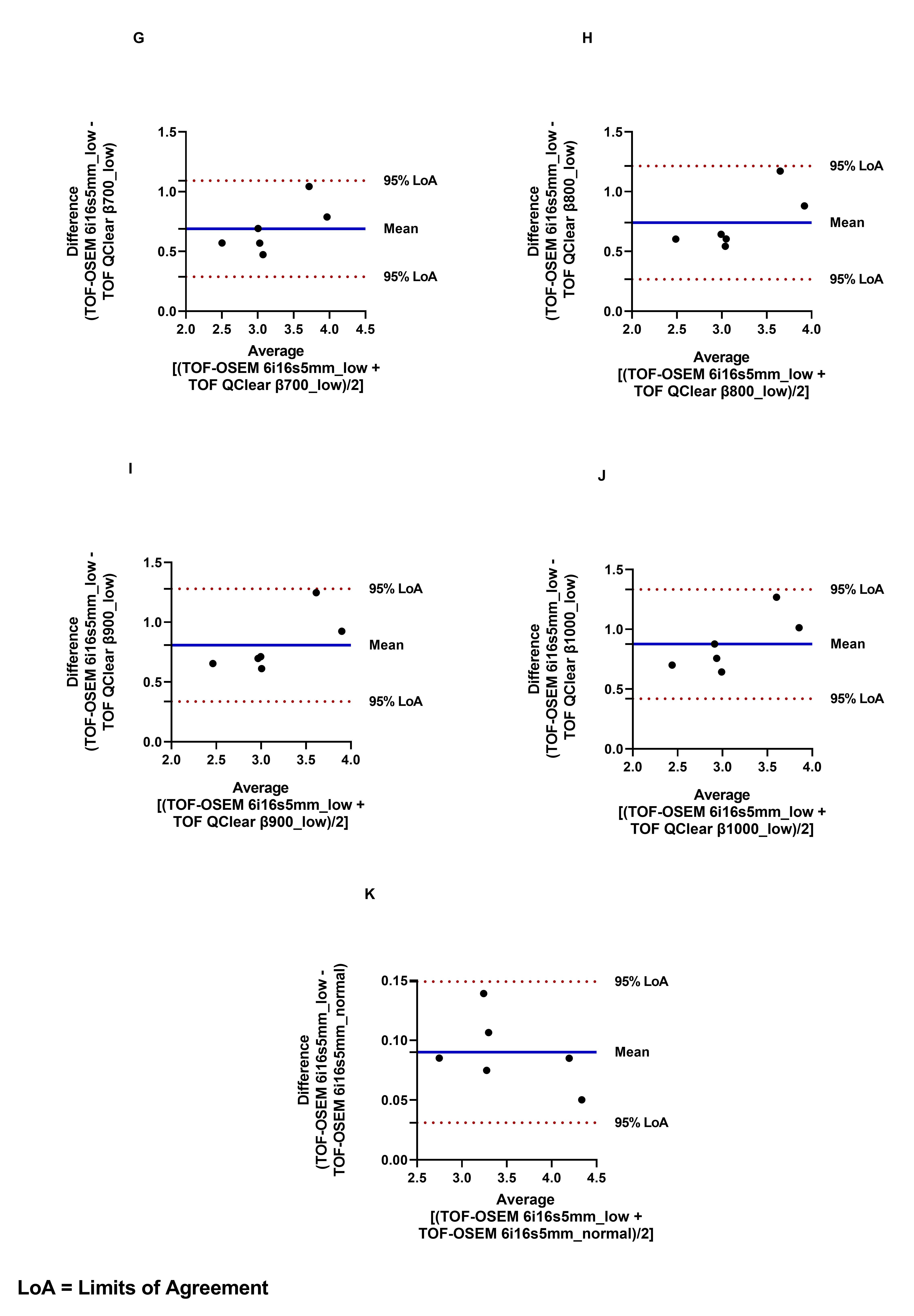

Supplement: Supplementary file 3 — Additional file 3. Figure S3: Bland-Altman plots of the BPND obtained for the Globus Pallidus: (G) TOF_OSEM 6i16s5mm _low vs TOF_Q.Clear β700_low; (H) TOF_OSEM 6i16s5mm _low vs TOF_Q.Clear β800_low; (I) TOF_OSEM 6i16s5mm_low vs TOF_Q.Clear β900_low; (J) TOF_OSEM 6i16s5mm _low vs TOF_Q.Clear β1000_low; (K) TOF_OSEM 6i16s5mm _low vs TOF_OSEM 6i16s5mm_normal. [file 13550_2022_883_MOESM3_ESM.jpg]

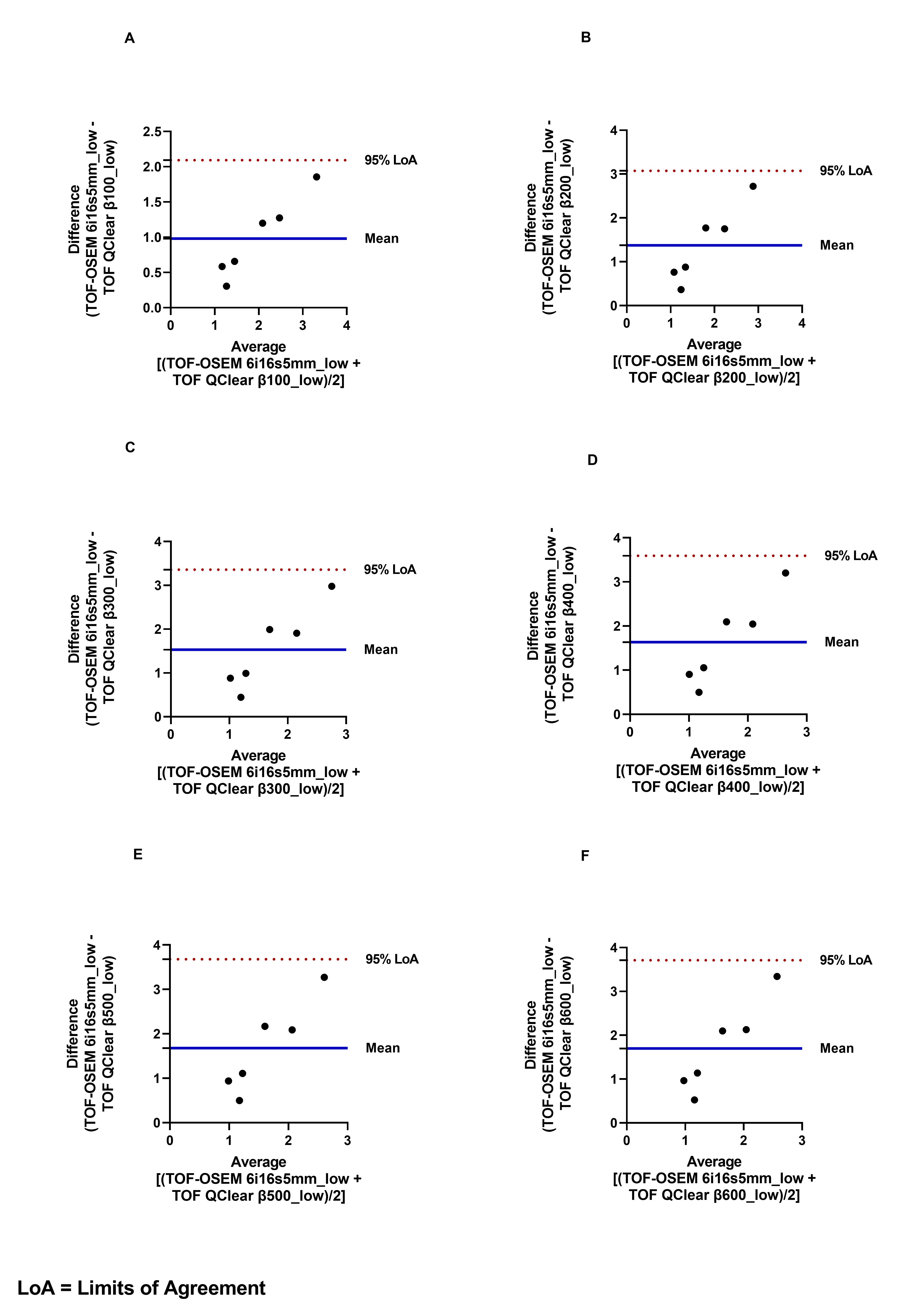

Supplement: Supplementary file 4 — Additional file 4. Figure S4: Bland-Altman plots of the BPND obtained for the Substantia Nigra: (A) TOF_OSEM 6i16s5mm_low vs TOF_Q.Clear β100_low; (B) TOF_OSEM 6i16s5mm _low vs TOF_Q.Clear β200_low; (C) TOF_OSEM 6i16s5mm_low vs TOF_Q.Clear β300_low; (D) TOF_OSEM 6i16s5mm _low vs TOF_Q.Clear β400_low; (E) TOF_OSEM 6i16s5mm_low vs TOF_Q.Clear β500_low; (F) TOF_OSEM 6i16s5mm_low vs TOF_Q.Clear β600_low. [file 13550_2022_883_MOESM4_ESM.jpg]

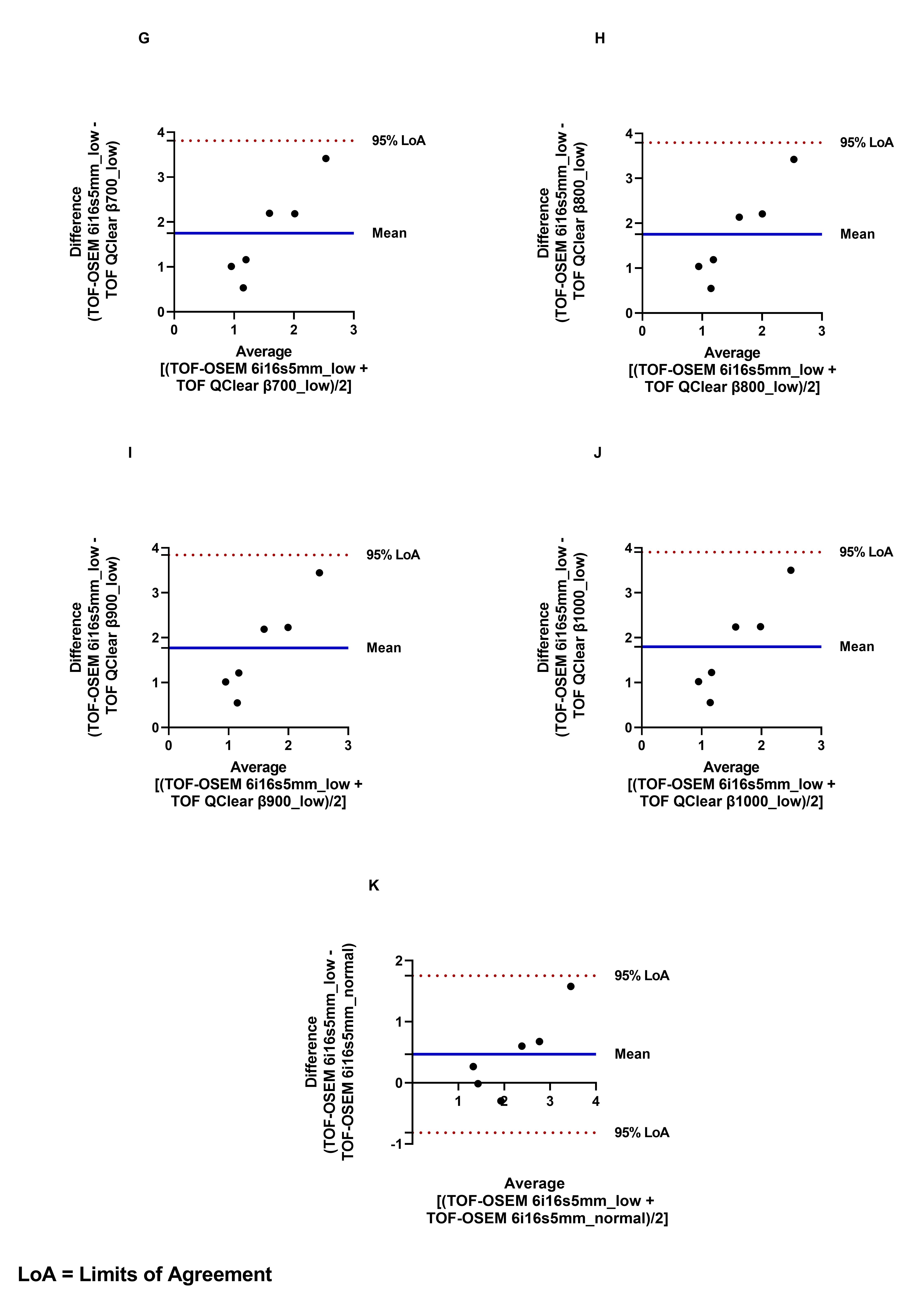

Supplement: Supplementary file 5 — Additional file 5. Figure S5: Bland-Altman plots of the BPND obtained for the Substantia Nigra: (G) TOF_OSEM 6i16s5mm _low vs TOF_Q.Clear β700_low; (H) TOF_OSEM 6i16s5mm _low vs TOF_Q.Clear β800_low; (I) TOF_OSEM 6i16s5mm_low vs TOF_Q.Clear β900_low; (J) TOF_OSEM 6i16s5mm _low vs TOF_Q.Clear β1000_low; (K) TOF_OSEM 6i16s5mm _low vs TOF_OSEM 6i16s5mm_normal. [file 13550_2022_883_MOESM5_ESM.jpg]
