# Supplementary material for: Assessing the impact of different penalty factors of the Bayesian reconstruction algorithm Q.Clear on in vivo low count kinetic analysis of [11C]PHNO brain PET-MR studies
Source: EJNMMI Res. 2022 Feb 20;12:11. doi: 10.1186/s13550-022-00883-1 (PMC8859021; doi:10.1186/s13550-022-00883-1)
Supplement: Supplementary file 6 — Additional file 6. Table S1. Bias, Standard deviation of Bias, Repeatability Coefficients (RC), Lower Limits of Agreement (LoA), Higher LoA, standard deviation of Bias and LoA obtained, per brain structure, when Q. Clear reconstructions with pre-frame delay and OSEM reconstruction with normal frame length were compared to standard OSEM reconstruction with pre-frame delay. [file 13550_2022_883_MOESM6_ESM.docx]

## Supplementary Table 1

**S.Table 1**

**S.Table 1** Bias, Standard deviation of Bias, Repeatability Coefficients (RC), Lower Limits of Agreement (LoA), Higher LoA,, standard deviation of Bias and LoA obtained, per brain structure, when Q.Clear reconstructions with pre-frame delay and OSEM reconstruction with normal frame length were compared to standard OSEM reconstruction with pre-frame delay.

|  |  | **Bias** | **SD Bias** | **RC** | **Lower LoA** | **Higher LoA** |
| --- | --- | --- | --- | --- | --- | --- |
| **SN** |  |  |  |  |  |  |
|  | TOF_OSEM6i16s5mm_low vs TOF_Q.Clear 100_low | 0.979 | 0.568 | 2.172 | -0.135 | 2.093 |
|  | TOF_OSEM6i16s5mm_low vs TOF_Q.Clear 200_low | 1.376 | 0.867 | 3.110 | -0.323 | 3.074 |
|  | TOF_OSEM6i16s5mm_low vs TOF_Q.Clear 300_low | 1.533 | 0.931 | 3.435 | -0.293 | 3.358 |
|  | TOF_OSEM6i16s5mm_low vs TOF_Q.Clear 400_low | 1.634 | 1.000 | 3.668 | -0.326 | 3.593 |
|  | TOF_OSEM6i16s5mm_low vs TOF_Q.Clear 500_low | 1.679 | 1.021 | 3.763 | -0.322 | 3.679 |
|  | TOF_OSEM6i16s5mm_low vs TOF_Q.Clear 600_low | 1.699 | 1.027 | 3.804 | -0.313 | 3.712 |
|  | TOF_OSEM6i16s5mm_low vs TOF_Q.Clear 700_low | 1.751 | 1.052 | 3.914 | -0.310 | 3.812 |
|  | TOF_OSEM6i16s5mm_low vs TOF_Q.Clear 800_low | 1.756 | 1.040 | 3.914 | -0.283 | 3.796 |
|  | TOF_OSEM6i16s5mm_low vs TOF_Q.Clear 900_low | 1.773 | 1.055 | 3.955 | -0.293 | 3.840 |
|  | TOF_OSEM6i16s5mm_low vs TOF_Q.Clear 1000_low | 1.798 | 1.075 | 4.015 | -0.308 | 3.904 |
|  | TOF_OSEM6i16s5mm_low vs TOF_OSEM6i16s5mm_normal | 0.469 | 0.656 | 1.490 | -0.817 | 1.754 |
|  |  |  |  |  |  |  |
| **St** |  |  |  |  |  |  |
|  | TOF_OSEM6i16s5mm_low vs TOF_Q.Clear 100_low | -0.093 | 0.063 | 0.213 | -0.215 | 0.030 |
|  | TOF_OSEM6i16s5mm_low vs TOF_Q.Clear 200_low | 0.046 | 0.056 | 0.135 | -0.062 | 0.155 |
|  | TOF_OSEM6i16s5mm_low vs TOF_Q.Clear 300_low | 0.162 | 0.036 | 0.324 | 0.092 | 0.232 |
|  | TOF_OSEM6i16s5mm_low vs TOF_Q.Clear 400_low | 0.213 | 0.065 | 0.432 | 0.085 | 0.340 |
|  | TOF_OSEM6i16s5mm_low vs TOF_Q.Clear 500_low | 0.285 | 0.044 | 0.565 | 0.198 | 0.372 |
|  | TOF_OSEM6i16s5mm_low vs TOF_Q.Clear 600_low | 0.344 | 0.036 | 0.678 | 0.274 | 0.414 |
|  | TOF_OSEM6i16s5mm_low vs TOF_Q.Clear 700_low | 0.378 | 0.044 | 0.745 | 0.293 | 0.464 |
|  | TOF_OSEM6i16s5mm_low vs TOF_Q.Clear 800_low | 0.432 | 0.045 | 0.851 | 0.343 | 0.521 |
|  | TOF_OSEM6i16s5mm_low vs TOF_Q.Clear 900_low | 0.468 | 0.054 | 0.921 | 0.361 | 0.574 |
|  | TOF_OSEM6i16s5mm_low vs TOF_Q.Clear 1000_low | 0.496 | 0.057 | 0.976 | 0.384 | 0.607 |
|  | TOF_OSEM6i16s5mm_low vs TOF_OSEM6i16s5mm_normal | 0.020 | 0.019 | 0.053 | -0.018 | 0.058 |
| **GP** |  |  |  |  |  |  |
|  | TOF_OSEM6i16s5mm_low vs TOF_Q.Clear 100_low | -0.087 | 0.077 | 0.220 | -0.239 | 0.065 |
|  | TOF_OSEM6i16s5mm_low vs TOF_Q.Clear 200_low | 0.116 | 0.112 | 0.303 | -0.103 | 0.335 |
|  | TOF_OSEM6i16s5mm_low vs TOF_Q.Clear 300_low | 0.295 | 0.202 | 0.682 | -0.100 | 0.691 |
|  | TOF_OSEM6i16s5mm_low vs TOF_Q.Clear 400_low | 0.405 | 0.144 | 0.834 | 0.123 | 0.687 |
|  | TOF_OSEM6i16s5mm_low vs TOF_Q.Clear 500_low | 0.521 | 0.178 | 1.069 | 0.172 | 0.869 |
|  | TOF_OSEM6i16s5mm_low vs TOF_Q.Clear 600_low | 0.593 | 0.217 | 1.226 | 0.167 | 1.019 |
|  | TOF_OSEM6i16s5mm_low vs TOF_Q.Clear 700_low | 0.690 | 0.205 | 1.401 | 0.288 | 1.091 |
|  | TOF_OSEM6i16s5mm_low vs TOF_Q.Clear 800_low | 0.741 | 0.242 | 1.515 | 0.268 | 1.215 |
|  | TOF_OSEM6i16s5mm_low vs TOF_Q.Clear 900_low | 0.808 | 0.241 | 1.641 | 0.336 | 1.279 |
|  | TOF_OSEM6i16s5mm_low vs TOF_Q.Clear 1000_low | 0.876 | 0.233 | 1.768 | 0.419 | 1.334 |
|  | TOF_OSEM6i16s5mm_low vs TOF_OSEM6i16s5mm_normal | 0.090 | 0.030 | 0.185 | 0.031 | 0.149 |
| **Th** |  |  |  |  |  |  |
|  | TOF_OSEM6i16s5mm_low vs TOF_Q.Clear 100_low | 0.002 | 0.045 | 0.080 | -0.086 | 0.090 |
|  | TOF_OSEM6i16s5mm_low vs TOF_Q.Clear 200_low | -0.021 | 0.074 | 0.138 | -0.166 | 0.124 |
|  | TOF_OSEM6i16s5mm_low vs TOF_Q.Clear 300_low | -0.021 | 0.083 | 0.155 | -0.184 | 0.143 |
|  | TOF_OSEM6i16s5mm_low vs TOF_Q.Clear 400_low | -0.037 | 0.077 | 0.155 | -0.187 | 0.114 |
|  | TOF_OSEM6i16s5mm_low vs TOF_Q.Clear 500_low | -0.047 | 0.099 | 0.200 | -0.241 | 0.148 |
|  | TOF_OSEM6i16s5mm_low vs TOF_Q.Clear 600_low | -0.048 | 0.095 | 0.194 | -0.234 | 0.138 |
|  | TOF_OSEM6i16s5mm_low vs TOF_Q.Clear 700_low | -0.069 | 0.105 | 0.231 | -0.274 | 0.136 |
|  | TOF_OSEM6i16s5mm_low vs TOF_Q.Clear 800_low | -0.068 | 0.107 | 0.232 | -0.277 | 0.141 |
|  | TOF_OSEM6i16s5mm_low vs TOF_Q.Clear 900_low | -0.068 | 0.115 | 0.246 | -0.294 | 0.158 |
|  | TOF_OSEM6i16s5mm_low vs TOF_Q.Clear 1000_low | -0.079 | 0.122 | 0.268 | -0.318 | 0.160 |
|  | TOF_OSEM6i16s5mm_low vs TOF_OSEM6i16s5mm_normal | 0.032 | 0.044 | 0.101 | -0.055 | 0.119 |
| **Cd** |  |  |  |  |  |  |
|  | TOF_OSEM6i16s5mm_low vs TOF_Q.Clear 100_low | -0.185 | 0.062 | 0.380 | -0.307 | -0.064 |
|  | TOF_OSEM6i16s5mm_low vs TOF_Q.Clear 200_low | -0.041 | 0.090 | 0.180 | -0.217 | 0.136 |
|  | TOF_OSEM6i16s5mm_low vs TOF_Q.Clear 300_low | 0.078 | 0.085 | 0.215 | -0.088 | 0.244 |
|  | TOF_OSEM6i16s5mm_low vs TOF_Q.Clear 400_low | 0.120 | 0.115 | 0.313 | -0.106 | 0.346 |
|  | TOF_OSEM6i16s5mm_low vs TOF_Q.Clear 500_low | 0.189 | 0.131 | 0.438 | -0.068 | 0.445 |
|  | TOF_OSEM6i16s5mm_low vs TOF_Q.Clear 600_low | 0.275 | 0.062 | 0.549 | 0.154 | 0.396 |
|  | TOF_OSEM6i16s5mm_low vs TOF_Q.Clear 700_low | 0.266 | 0.150 | 0.585 | -0.028 | 0.559 |
|  | TOF_OSEM6i16s5mm_low vs TOF_Q.Clear 800_low | 0.339 | 0.098 | 0.686 | 0.147 | 0.531 |
|  | TOF_OSEM6i16s5mm_low vs TOF_Q.Clear 900_low | 0.377 | 0.119 | 0.769 | 0.143 | 0.611 |
|  | TOF_OSEM6i16s5mm_low vs TOF_Q.Clear 1000_low | 0.372 | 0.174 | 0.792 | 0.031 | 0.712 |
|  | TOF_OSEM6i16s5mm_low vs TOF_OSEM6i16s5mm_normal | 0.010 | 0.033 | 0.062 | -0.054 | 0.074 |
| **Pt** |  |  |  |  |  |  |
|  | TOF_OSEM6i16s5mm_low vs TOF_Q.Clear 100_low | -0.090 | 0.060 | 0.208 | -0.209 | 0.028 |
|  | TOF_OSEM6i16s5mm_low vs TOF_Q.Clear 200_low | 0.015 | 0.054 | 0.100 | -0.090 | 0.120 |
|  | TOF_OSEM6i16s5mm_low vs TOF_Q.Clear 300_low | 0.119 | 0.021 | 0.236 | 0.077 | 0.160 |
|  | TOF_OSEM6i16s5mm_low vs TOF_Q.Clear 400_low | 0.151 | 0.053 | 0.310 | 0.048 | 0.254 |
|  | TOF_OSEM6i16s5mm_low vs TOF_Q.Clear 500_low | 0.226 | 0.037 | 0.447 | 0.153 | 0.298 |
|  | TOF_OSEM6i16s5mm_low vs TOF_Q.Clear 600_low | 0.278 | 0.037 | 0.549 | 0.206 | 0.351 |
|  | TOF_OSEM6i16s5mm_low vs TOF_Q.Clear 700_low | 0.315 | 0.045 | 0.622 | 0.226 | 0.404 |
|  | TOF_OSEM6i16s5mm_low vs TOF_Q.Clear 800_low | 0.356 | 0.031 | 0.701 | 0.295 | 0.418 |
|  | TOF_OSEM6i16s5mm_low vs TOF_Q.Clear 900_low | 0.399 | 0.036 | 0.784 | 0.329 | 0.469 |
|  | TOF_OSEM6i16s5mm_low vs TOF_Q.Clear 1000_low | 0.435 | 0.051 | 0.858 | 0.336 | 0.534 |
|  | TOF_OSEM6i16s5mm_low vs TOF_OSEM6i16s5mm_normal | 0.034 | 0.027 | 0.081 | -0.018 | 0.086 |
