# Supplementary material for: Assessing the impact of different penalty factors of the Bayesian reconstruction algorithm Q.Clear on in vivo low count kinetic analysis of [11C]PHNO brain PET-MR studies
Source: EJNMMI Res. 2022 Feb 20;12:11. doi: 10.1186/s13550-022-00883-1 (PMC8859021; doi:10.1186/s13550-022-00883-1)
Supplement: Supplementary file 7 — Additional file 7. Table S2. Coefficient of variation obtained for the Substantia Nigra, Striatum, Globus Pallidus, Thalamus, Caudate and Putamen, per reconstruction Method. Note the highest percentages are observed for the Substantia Nigra and Thalamus. [file 13550_2022_883_MOESM7_ESM.docx]

## Supplementary Table 2

**S.Table 2**

**S.Table 2** Coefficient of variation obtained for the Substantia Nigra, Striatum, Globus Pallidus, Thalamus, Caudate and Putamen, per reconstruction Method. Note the highest percentages are observed for the Substantia Nigra and Thalamus.

| **Reconstruction Method** | **%CV Substantia Nigra** | **%CV Striatum** | **%CV Globus Pallidus** | **%CV Thalamus** | **%CV Caudate** | **%CV Putamen** |
| --- | --- | --- | --- | --- | --- | --- |
| **1_ TOF _OSEM_6i16s5mm_low** | 45.41832493 | 11.72287022 | 17.1263507 | 19.75959975 | 14.93303633 | 11.65408554 |
| **2_ TOF _QClear_B100_low** | 38.17577226 | 12.84491396 | 17.89216232 | 14.17978715 | 15.58405133 | 12.61996998 |
| **3_ TOF _QClear_B200_low** | 28.72130884 | 12.75007419 | 17.45920353 | 25.74974894 | 16.46186062 | 12.69296747 |
| **4_ TOF _QClear_B300_low** | 30.28512922 | 12.33280212 | 16.05931007 | 30.63244265 | 15.28267088 | 12.15481101 |
| **5_ TOF _QClear_B400_low** | 27.32815403 | 12.97046284 | 16.59931675 | 26.94987759 | 17.09950166 | 12.68228813 |
| **6_ TOF _QClear_B500_low** | 29.60766734 | 13.00523057 | 16.30889312 | 31.5792602 | 17.01502152 | 12.77713343 |
| **7_ TOF _QClear_B600_low** | 26.63014208 | 13.21577293 | 15.45279731 | 31.18768195 | 16.95801749 | 12.91191471 |
| **8_ TOF _QClear_B700_low** | 29.61208195 | 13.36629606 | 16.21458635 | 31.22143308 | 17.23967698 | 13.14849772 |
| **9_ TOF _QClear_B800_low** | 28.54199913 | 13.13942075 | 15.27243218 | 32.27131464 | 15.55622549 | 12.80121683 |
| **10_ TOF _QClear_B900_low** | 29.02665991 | 13.15239998 | 15.75640103 | 33.87790044 | 16.49121518 | 13.07511373 |
| **11_ TOF _QClear_B1000_low** | 30.17244768 | 13.45800577 | 16.1175274 | 34.42014471 | 18.24451088 | 13.31993369 |
| **12_ TOF _OSEM_6i16s5mm_normal** | 28.61458868 | 11.49902568 | 17.99716144 | 20.76490227 | 14.57108139 | 11.84132012 |
